# The Impact of Freeze-Dried *Tenebrio molitor* Larvae on the Quality, Safety Parameters, and Sensory Acceptability of Wheat Bread

**DOI:** 10.3390/insects15080603

**Published:** 2024-08-10

**Authors:** Agnė Jankauskienė, Aistė Kabašinskienė, Dominykas Aleknavičius, Sandra Kiseliovienė, Sigita Kerzienė, Vytautė Starkutė, Elena Bartkienė, Monika Zimkaitė, Ignė Juknienė, Paulina Zavistanavičiūtė

**Affiliations:** 1 Department of Food Safety and Quality, Veterinary Academy, Lithuanian University of Health Sciences, Tilzes St. 18, LT-47181 Kaunas, Lithuaniaelena.bartkiene@lsmu.lt (E.B.);; 2 Divaks, Private Limited Company (UAB), Vinco Kudirkos g. 22-12, LT-01113 Vilnius, Lithuania; 3 Food Institute, Kaunas University of Technology, Radvilenu pl. 19, LT-50254 Kaunas, Lithuania; 4 Department of Physics, Mathematics and Biophysics, Lithuanian University of Health Sciences, Veterinary Academy, Tilzes St. 18, LT-47181 Kaunas, Lithuania; 5 Institute of Animal Rearing Technologies, Lithuanian University of Health Sciences, Tilzes Str. 18, LT-47181 Kaunas, Lithuania

**Keywords:** lyophilization, nutritional value, amino, fatty acids, sugars, acrylamide, acceptability, mealworms, sustainability

## Abstract

**Simple Summary:**

In this study, we investigated the impact of incorporating freeze-dried mealworm larvae powder (*Tenebrio molitor*) into wheat bread on its quality, nutritional value, and consumer acceptance. Mealworms represent a sustainable protein source that can enhance the nutritional profile of foods. Bread was prepared with varying concentrations of mealworm powder (5%, 10%, and 15%), which significantly increased the bread’s protein and fat content, particularly in terms of essential amino acids and beneficial fatty acids. However, higher levels of mealworm powder also resulted in darker bread and decreased consumer acceptance, as the altered taste and texture were perceived less favourably. This study emphasizes that while mealworm powder can enhance the nutritional value of bread, it is crucial to balance these benefits with sensory qualities to maintain consumer appeal. The findings suggest that mealworm powder could be a valuable ingredient for producing nutritionally enriched bread, provided that consumer preferences are carefully considered. This research contributes to the growing body of literature on the use of insects as sustainable food ingredients.

**Abstract:**

The research context involves analyzing the potential benefits derived from integrating insect protein into everyday food items. Utilizing methods consistent with established food science protocols, wheat bread was prepared with variations of 0%, 5%, 10%, and 15% *Tenebrio molitor* larvae powder, derived from larvae cultivated on brewery spent grain. A substrate selected for its superior nutritional content and a substrate with agar–agar gels were used. The tests included basic bread tests; sugar, acrylamide, amino, and fatty acid (FA) tests; and sensory acceptability. The results have shown that the acrylamide levels in bread with larvae remained below harmful thresholds, suggesting that using *T. molitor* can be a safe alternative protein source. The incorporation of powdered *T. molitor* larvae (p-TMLs) into bread was observed to increase certain sugar levels, such as glucose, particularly at higher larval concentrations. The addition of *T. molitor* significantly raised the protein and fat levels in bread. The inclusion of larvae enriched the bread with essential amino acids, enhancing the nutritional value of the bread significantly. The FA profile of the bread was altered by the inclusion of p-TMLs, increasing the levels of monounsaturated FAs. Despite the nutritional benefits, higher concentrations of larvae decreased the sensory acceptability of the bread. This suggests that there is a balance to be found between enhancing the nutritional content and maintaining consumer appeal. These findings highlight the potential for using p-TMLs as a sustainable, nutritious ingredient in bread making, although the sensory qualities at higher concentrations might limit consumer acceptance.

## 1. Introduction

The global food industry is steadily integrating sustainable and unconventional protein sources to address the rising demand for food and the environmental impact of traditional livestock farming [1]. Among alternative protein sources, insects, particularly *Tenebrio molitor* (mealworm) larvae, are gaining attention due to their high nutritional value and lower ecological footprint [2]. Insect-based food ingredients have been noted for their high protein, fat, and micronutrient contents, presenting a viable supplementation solution in human diets [3]. For this reason, the utilization of *T. molitor* in food products has been studied primarily for its potential to enhance nutritional benefits [4]. Recent studies indicate that the addition of p-TMLs can affect the nutritional profiles of food products, increasing the levels of essential amino acids and altering the FA compositions [5,6]. Previous research has predominantly focused on the use of mealworms in snack foods and pasta or bread, with studies highlighting the potential to improve their acceptance in the Western world and reduce ecological impacts [7]. Mealworms were also incorporated into tortillas, leading to an enhanced protein content, as noted by Aguilar et al. [8] Additionally, replacing 10% of lean pork with mealworms in frankfurters achieved a quality level comparable to that of standard products [9,10]. These findings support the potential of mealworms as a viable ingredient in human food.

Previous research has highlighted the potential for insect protein to not only enhance nutritional profiles—by boosting the protein, amino acid, and FA levels—but also to influence the physical and sensory qualities of food products [2,11,12,13]. However, Western societies may initially resist adopting insects as a protein source due to their absence from traditional Western diets [14]. The integration of insect proteins into widely consumed foods like bread poses challenges, particularly regarding consumer acceptance and the modification of traditional food textures and flavors [15,16,17]. Despite the potential benefits, the sensory acceptability of insect-enriched foods varies significantly, necessitating careful formulation to balance taste preferences and cultural perceptions, which currently hinder acceptance in Western cultures [18,19,20,21]. A key area of contention in this field revolves around the optimal level of insect protein inclusion that balances nutritional enhancement with sensory acceptability. Researchers found that consumers are more inclined to consume insects in less visible forms, such as powder, which simplifies their incorporation into food products [22]. However, studies have shown varying consumer responses based on the concentration of insect ingredients, with higher levels often leading to decreased sensory appeal [23,24,25,26]. Consumers found muffins containing 8% mealworm to be acceptable, according to Hwang and Choi’s study [27].

Zielińska and Pankiewicz note that cereal-based products, including bread, biscuits, and other bakery items, enjoy widespread popularity and acceptance globally [28]. Bread, a staple food made from ingredients like wheat flour, water, salt, and yeast, is a fundamental part of the global diet primarily because it is a rich source of carbohydrates and serves as a significant energy source, as described by de Oliveira and Osimani et al. [29,30]. Adding p-TMLs to bread can enhance its nutritional profile by significantly increasing the protein and essential amino acid contents, offering a richer and more balanced nutritional composition compared to traditional bread [31,32]. This enhancement could be particularly beneficial for populations with higher protein needs, such as athletes, elderly individuals, and people with specific dietary deficiencies or restrictions, providing them with a more complete source of essential nutrients. The incorporation of p-TMLs also leads to higher levels of unsaturated FAs, particularly omega-3 and omega-6, which are beneficial for cardiovascular health [33]. Schösler et al. highlight that disguising insects in food products by incorporating them in a form that makes them unrecognizable, such as powders, can enhance consumer acceptance [34]. This approach leverages the fact that visual appeal is critical in shaping initial consumer perceptions, suggesting that less visible forms of insects could facilitate their acceptance in the Western diet. Additionally, there are concerns about food safety parameters such as the sugar and acrylamide levels, which could be influenced by the type and concentration of insect-derived ingredients [34,35,36].

However, the incorporation of freeze-dried p-TMLs into wheat bread presents a novel context for investigation, particularly in terms of how it affects the bread quality and safety parameters. This research contributes to the broader discourse on sustainable food sources by providing empirical data on the use of insect protein in everyday food items like bread. It addresses the gap in understanding the dual implications of such fortification, not only in enhancing the nutritional content but also in maintaining or improving sensory and safety aspects to ensure market viability. The findings are expected to offer insights into the optimal use of *T. molitor* in bread making, balancing nutritional benefits with consumer preferences.

## 2. Materials and Methods

### 2.1. Materials Used for Bread Preparation

In the experiment for preparing wheat bread (WB), wheat flour (type 812C) characterized by a falling number of 315 s, gluten content of 30%, and ash content of 0.74% was utilized. This flour was sourced from Kauno Grūdai Ltd. mill located in Kaunas, Lithuania [37]. The WB samples were crafted both without any additions and with varying concentrations (5%, 10%, and 15%) of p-TMLs.

### 2.2. Insect Cultivation and Mealworm Powder Preparation

The choice of substrates and cultivation conditions for mealworms was informed by earlier studies, with a preference for larvae cultivated on dehydrated brewer’s spent grain. This preference was due to its superior content of trace elements, higher protein levels, optimal sensory ratings (apart from the control), the greatest fiber content, and the most favorable FA and amino acid profiles. The control group of mealworm larvae used for comparison was grown on agar–agar gels (for sensory analyses), which was particularly well evaluated during the sensory analysis in our previous studies [31,32].

*T. monitor* larvae were cultivated using brewery spent grains under analogous conditions, as in our previously published articles (Table 1) [10,31,32,38]. The yellow mealworm larvae were raised under controlled conditions at the Divaks company’s insect research and development facility in Vilnius, Lithuania [39], maintaining a temperature of 27 ± 2 °C, humidity of 60 ± 5%, and providing lighting for no more than 1 h per day (limiting light exposure to the time needed for operators to work) to achieve optimal growth and reduce stress. Wheat bran from Fasma, Lithuania (Fasma, Radviliškis, Lithuania) [40], were used as the primary substrate for adult beetles of various ages. Approximately 30,000 individuals were placed in containers with 3.45 kg carrots for moisture from Sanitex, Lithuania (Sanitex, Kaunas, Lithuania) [41] (provided three times a week), and 1.5 kg of dry feed consisting of dehydrated brewer’s spent grain and brewer’s yeast from Ekoproduktas, Panevėžys, Lithuania [42], with a ratio of 9:1 (totaling 4 kg during this period). Another experimental group was cultured on agar–agar (10 g/L) gels (Carl Roth, Darmstadt, Germany), and as the dry feed, we used wheat bran at a 9:1 ratio with brewer’s yeast. The larvae were considered fully grown upon the appearance of the first pupae, after 56 days of growth, followed by a 24 h fasting period in a climate chamber before being processed and frozen at −18 °C for the subsequent analysis.

All mealworms were dried in a thermal oven at 103 °C until they reached a constant mass, while another portion underwent rapid freezing at −35 °C for 8 h using a Liebherr fast freezer (LGv 5010 MediLine, Richmond, BC, Canada). Freeze drying was performed in a lyophilizer (Harvest Right, North Salt Lake, UT, USA) until reaching 80 °C under a pressure of 73 (Pa), lasting a total of 72 h. The lyophilized and dried larvae were then milled using a laboratory-scale (Fritsch Mill Pulverisette 14, Idar-Oberstein, Germany) mill at 6000 rpm.

### 2.3. Bread Preparation

WB preparation was analogous to the method described by Bartkienė et al. in their study [43]. For the preparation of the WB samples, the formula included 1 kg of wheat flour, 1.5% salt, 2% fresh compressed yeast, and 56% water, which served as the control bread (Table 2). These control WB (WB-C) samples did not incorporate any mealworm powder. We tested p-TMLs at 5%, 10%, and 15% to evaluate the effects and benefits of higher inclusion rates. The European Commission limits insect meal use to 10%, but our aim was to assess the sensory and nutritional properties at varying levels, including potential diminishing returns or adverse effects beyond the regulatory limit. Initially, the dough was mixed using a KitchenAid Artisan mixer (Greenville, OH, USA) for 3 min at low speed followed by 7 min at high speed. Subsequently, the dough underwent a relaxation period of 12 min at a temperature of 22 ± 2 °C. Afterward, the dough was formed into loaves, shaped, and allowed to proof in conditions of 30 ± 2 °C and 80% relative humidity for 60 min. Finally, the loaves were baked at 220 °C for 25 min in a deck oven manufactured by EKA (Borgoricco, Italy).

### 2.4. Evaluation of Bread Quality Parameters

Following a 12 h cooling period at a temperature of 22 ± 2 °C, the WB samples underwent evaluations for the specific volume, crumb porosity, shape coefficient, mass loss post-baking, and color coordinates of both the crust and crumb.

#### 2.4.1. Mass Loss Post-Baking

The mass loss following baking was quantified as a percentage by comparing the mass of the loaf dough before and after baking.

#### 2.4.2. Porosity

This method involves analyzing the structure of the bread crumb to determine the air pockets and their distribution. The porosity of the bread crumb was assessed according to the LST method 1442:(1996) [44].

#### 2.4.3. Shape Coefficient

The coefficient of the bread shape was determined by calculating the ratio of the width to the height of the bread slice, measured in millimeters.

#### 2.4.4. Specific Bread Volume

The bread volume was determined using the AACC method [45], with the specific volume derived from the volume-to-weight ratio. In this procedure, the bread loaf was placed in a measuring container, which was then filled with millet grains to measure the displaced volume. The specific volume is calculated as the ratio of the bread’s volume to its weight.

#### 2.4.5. Color Coordinates

The color parameters of crust and crumb WB samples were evaluated using a Chromameter CR-400 (Konica Minolta, Marunouchi, Japan) in reflection mode. The color parameters evaluated included L* (lightness), a* (redness), and b* (yellowness), and the evaluations were conducted using a D65 light source, a 2° observer angle, and an 8 mm aperture diameter.

### 2.5. Nutritional Value

The tests of nutritional value parameters were carried out in an accredited laboratory: the Chemical Science Laboratory, Food Institute, Kaunas University of Technology, Lithuania [46].

#### 2.5.1. Amino Acid Content

The amino acid compositions of the samples were analyzed via ultrafast liquid chromatography (UFLC) with automated o-phthalaldehyde and 9-fluorenylmethyl chloroformate (FMOC)/Mercaptopropionic Acid derivatization. Standard solutions of the amino acids, including alanine, aspartic acid, arginine, cystine, glycine, valine, leucine, isoleucine, threonine, serine, proline, methionine, glutamic acid, phenylalanine, lysine, histidine, tyrosine, asparagine, and tryptophan, were used for this analysis (A9781 Sigma-Aldrich, Steinheim, Germany) [47]. To commence the analysis, each sample (approx. 0.4 g) underwent hydrolysis with 25 mL of 6 M HCl for 24 h at 103 °C. The resultant contents were quantitatively transferred into a 250 mL beaker using a 150–200 mL solution of 0.2 mol Na^+^/L and pH 2.20 trisodium citrate dihydrate. The resulting hydrolysate was partially neutralized by the gradual addition of 17 mL of 7.5 N sodium hydroxide solution while stirring continuously, ensuring the temperature remained below 40 °C (in a cold water bath). The pH was adjusted to 2.20 at room temperature using sodium hydroxide solution (7.5 N). Before injection, all samples were filtered through 0.45 μm filters. The amino acids were separated using the UHPLC column YMC-Triart C18 (1.9 μm, YMC Co., Ltd., Allentown, PA, USA) on a UFLC instrument (Shimadzu, Kyoto, Japan), which was equipped with a fluorescence detector RF-20Axs and a pre-treatment function-equipped automatic injector SIL-30AC (Shimadzu, Kyoto, Japan). The analytical conditions were as follows: a mobile phase consisting of solvent A (20 mmol/L potassium phosphate buffer, pH 6.5) and solvent B (45/40/15 acetonitrile/methanol/water); a flow rate set at 0.5 mL/min; a column temperature maintained at 45 °C; and detection wavelengths as follows: RF-20Axs Ex. at 350 nm, Em. at 450 nm to Ex. at 266 nm, and Em. at 305 nm (9.0 min). A calibration set comprising five levels was utilized, covering a concentration range of 9.375–150.00 μmol/L with the exception of cystine, which covered a concentration range of 8.08–75.00 μmol/L.

#### 2.5.2. Method for Determination of FAs

The analysis for the identification and quantification of FAs was conducted via gas chromatography utilizing a capillary column and flame-ionization detection. Initially, FAs were extracted from a 2 g sample using 15 mL of n-hexane (Chempur, Piekary Śląskie, Poland), followed by methylation with anhydrous KOH methanol solution to yield methyl esters, following the protocol outlined in ISO 12966–2:2013 [48]. The analysis of FA methyl esters was carried out using a Shimadzu GC-2010 gas chromatograph (Shimadzu, Kyoto, Japan) equipped with a flame ionization detector and a 100 m column (Restek Rt-2560) with a diameter of 0.25 µm and thickness of 0.20 µm, as specified in ISO 12966-4:2015 [49]. Chromatographic peaks were identified by comparing the retention times with a mixture of Supelco 37 Component FAME Mix reagent kit (Supelco Analytical, Bellefonte, PA, USA). The analytical conditions were as follows: a volume of 1 µL was injected, the column temperature was initially set at 100 °C for 4 min, and it was then ramped up to 240 °C at a rate of 13 °C/min and maintained for 63 min. The injector temperature was set at 250 °C and the detector temperature at 300 °C. Nitrogen was employed as the carrier gas.

#### 2.5.3. Method for Determination of Sugars in Bread Samples

The content of sugars was analyzed using an analogous method, as in the previously published article by Jankauskienė et al. [31].

#### 2.5.4. Method for Determination of Protein Content

The amount of protein in breads was determined according to the ISO standard 1871:2009, which provides general food and feed product guidelines for the determination of nitrogen content using the Kjeldahl method. The factor of conversion used was 6.25 [50].

#### 2.5.5. Method for Determination of Fat Content

The fat content in bread was calculated according to AOAC 922.06+AOAC 963.15:2003, p.31871:2009 [51,52,53].

### 2.6. Safety Parameters: Acrylamide Content in Bread

The acrylamide concentration was determined according to the method of Zhang et al. [54].

### 2.7. Analysis of Product Acceptability

**Figure 1:**
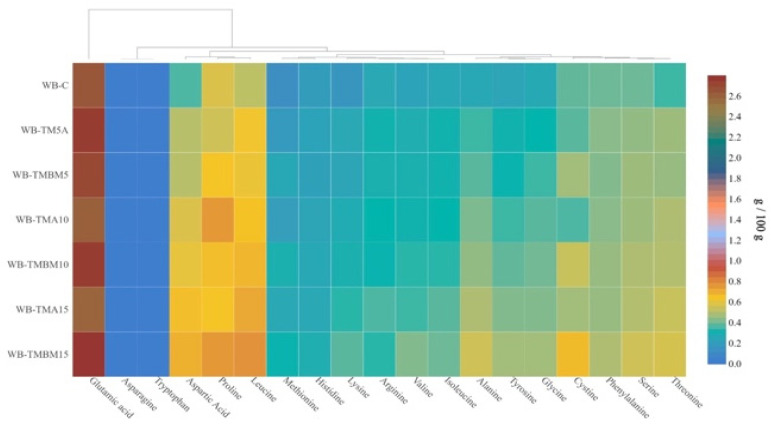
Amino acid compositions in bread samples enriched with freeze-dried p-TMLs, g/100 g.

The sensory characteristics of bread slices were assessed by 10 judges using a rating scale from 0 (extremely dislike) to 100 (extremely like), according to ISO method 8586:2023 [55]. Using similar software to that which was tested by Bartkienė et al. [43], bread tasting samples fortified with freeze-dried p-TMLs were assessed. The acceptability of the bread slices was evaluated by 10 consumers by analyzing their facial expressions using FaceReader 8.0 software (Noldus Information Technology, Wageningen, the Netherlands; see Figure 1), which analyzed facial expressions corresponding to eight emotional states (neutral, happy, sad, angry, surprised, scared, disgusted, and contempt) [56]. The bread samples were sequentially tasted in front of a Microsoft LifeCam Studio webcam (Microsoft Corporation, Redmond, WA, USA), which measured the intensity of the facial expressions on a scale from 0 to 1 and valence from −1 to 1. Consumers rinsed their mouths with water between samples.

### 2.8. Statistical Analysis

Statistical analysis was performed utilizing IBM SPSS Statistics 29.0.0.0 (241). The means and standard deviations of the variables investigated in the different groups were computed. The group differences were assessed through ANOVA with post hoc Bonferroni testing. Statistical significance was determined at a threshold of *p* < 0.05. The entire experiment was repeated three times.

## 3. Results and Discussion

The WB quality parameters (mass loss after baking, porosity, shape coefficient, and specific volume) and the influence of the analyzed factors (different quantities and types of freeze-dried and milled *T. molitor*) and their interactions are given in Table 3. The WB samples’ mass loss after baking was, on average, 9.9%. The highest (11.2%) mass loss after baking was found in the WB-TMBM15 sample. Different quantities and types of freeze-dried and milled *T. molitor* did not have a significant influence on this parameter (*p* = 0.465 and *p* = 0.955, respectively); however, the interaction of both factors was significant (*p* = 0.001) on the samples’ mass loss after thermal treatment.

There are no consistent trends in the literature regarding the porosity of WB with alternative insect and mealworm powders. Comparing the bread sample porosities, we found that the addition of 5% of both milled and freeze-dried *T. molitor* did not have a significant influence when compared to the results with control samples. For instance, Bartkiene et al. [43] found that 5% *Acheta domesticus* powder did not affect the porosity of the tested WB samples; however, by increasing the amount of these powder, the porosity decreased, respectively. We found the same tendencies, that 10 and 15% of both milled and freeze-dried *T. molitor* decrease the porosity, on average, by 13% compared with WB-C samples. However, according to da Rosa Machado and Thys, the incorporation of *Gryllus assimilis* powder (at 10% and 20%, both with and without oil) resulted in breads exhibiting an increased porosity. These results can be explained by the high protein and fat contents of the insect powder. Also, the protein’s physicochemical properties, such as its structure, solubility, and hydration, can affect bread’s porosity [57]. In addition to this, multivariate analysis showed that different quantities and types of freeze-dried milled *T. molitor* and their interactions have a significant (*p* = 0.001; *p* = 0.001, and *p* = 0.004, respectively) influence on WB’s porosity. We found that milled and freeze-dried *T. molitor* larvae grown on a brewer’s spent grain medium lowered the WB porosity, on average, by 5.5% when comparing the results with samples prepared with the same additive but grown on agar–gar gel (TMA).

Increasing the quantities of both freeze-dried and milled *T. molitor* types in WB formulations resulted in greater shape coefficients except in the WB-TMBM15 samples. However, the shape coefficient of WB-TMBM15 samples was higher by 26% when compared with control samples. Only the WB-TM5A and WB-C samples’ shape coefficients were the same, on average, at 1.465. WB samples with different quantities of TMBM had a higher shape coefficient by 1.16 times when compared to the results of samples made with TMA. Similar tendencies were found by Bartkiene et al., who claimed that adding 5 to 15% *A. domesticus* powder increased the WB shape coefficient, on average, by 34% [43]. According to multivariate analysis results, the types (freeze-dried and milled) of *T. molitor* and different quantities used and their interactions had a significant (*p* = 0.001, *p* = 0.001, and *p* = 0.001, respectively) influence on the WB shape coefficient. Also, a negative moderate correlation was established between the WB samples’ mass loss after baking and the shape coefficient as well as between the shape coefficient and porosity (r = −0.598, *p* = 0.001; and r = 0.501, *p* = 0.004, respectively).

The WB samples’ specific volume was not statistically different between all the tested samples, and results, on average, of 1.66 cm^3^ g^−1^ were found. The specific volume is an important bread quality attribute that impacted by the nutritional aspects and sensory parameters of bread loaves. In our case, lyophilized and milled *T. molitor* did not affect the specific volume of WB, which may be attributed to the higher fat content, because during the baking process, the melting fat stabilizes the expanding gas cells [58]. The changes in the specific volume of loaves of bread depend on the protein content of the alternative powder from edible insects or worms used. Proteins are known to interfere with the proper development of dough during fermentation, leading to a low gas retention capacity due to a weakened gluten network. This effect is particularly noticeable in bread made with more than 15% *Alphitobius diaperinus* and *A. domesticus* powders [43,58,59].

The bread production process is sensitive to the substitution of wheat flour, particularly with gluten-free and non-starch additives, as these replacements can disrupt the development of the gluten network. Our studies showed that the addition of freeze-dried and *milled T. molitor* had an ambiguous impact on the tested bread quality parameters.

Acrylamide is a chemical compound that can form during food cooking processes, especially when the foods are prepared using high temperatures, such as through baking or frying [60]. Acrylamide typically originates from sugars and the amino acid asparagine when food is heated above 120 °C [61]. Acrylamide is classified as a carcinogen (class 2A carcinogen), and prolonged exposure has been associated with neurotoxicity, reproductive toxicity, genotoxicity, developmental toxicity, and endocrine disruption, highlighting the need to minimize exposure to this harmful compound. [62,63,64].

Acrylamide, demonstrated in animal experiments to have carcinogenic effects, can induce cancerous tumors and nerve damage and is also genotoxic, indicating that it can damage DNA and contribute to mutations and cancer develop. Although a direct link between dietary acrylamide intake and cancer in humans has not been conclusively proven, the health effects of this substance are considered potentially hazardous.

According to Commission Regulation (EU) 2017/2158 [65], the acrylamide content in bread should not exceed 50 µg/kg. All the bread samples listed, including those with varying concentrations of *T. molitor* larvae, show acrylamide levels significantly below this threshold, ranging from 21.2 ± 0.02 to 30.4 ± 0.01 µg/kg for WB-TMBM15 (Table 4). There does not appear to be a consistent statistical trend regarding the concentration of larvae and the acrylamide levels; however, the highest value corresponds to the sample with 15% larvae grown on brewer’s spent grain (WB-TMBM15). Burešová et al. conducted a study investigating how the incorporation of different concentrations (5%, 8%, and 12%) of insect powder, specifically from field *A. domesticus* and yellow mealworms, affects acrylamide formation in both leavened and unleavened breads [66]. The addition of *A. domesticus* and *T. molitor* powder to wheat bread affects acrylamide formation by increasing the contents of reducing sugars and free amino acids while reducing asparagine, a key precursor in acrylamide formation in cereal products. While unleavened bread showed an increase in its acrylamide levels with higher insect supplementation compared to the control, leavened bread demonstrated a decreased acrylamide content with the highest insect supplementation (with *A. domesticus* at 64.84 and mealworm at 68.78 vs. the control sample at 82.47 µg/kg). In our study, asparagine was not detected in any of the samples, including the control group (Appendix A). This could have been one of the main reasons why our study found the acrylamide levels to be particularly low compared to other researchers’ studies, where other edible insects were incorporated, and asparagine was detected. Overall, the study suggests that enriching bakery products with insect powder can enhance their nutritional value without increasing the risk of acrylamide intake for consumers [66]. The EFSA Panel on Nutrition, when presenting its opinion on the formation of acrylamide, obtained completely different results than the aforementioned authors [67]. In the study, acrylamide formation was specifically investigated in biscuits containing p-TMLs; the biscuits were baked at a high temperature of 200 °C for 10 to 12 min. The study reported that the acrylamide concentration in these biscuits after baking was measured at 252 µg/kg. The results, according to the EFSA’s opinion, do not exceed the regulated amount (for biscuits, it must not exceed 350 µg/kg). The biscuit’s type of flour and the sugars added to cookies could have also influenced the higher formation of acrylamide [63]. This research highlights the importance of monitoring acrylamide levels when using insect-derived ingredients in food products to ensure that they meet safety standards [67].

The L* value (lightness) generally decreases (darker crust) with an increased p-TML concentration, which especially noticeable in WB-TMA10 (49.7 ± 0.8) and WB-TMBM5 (49.6 ± 0.4). The control (WB-C) shows a lighter crumb (74.6 ± 0.6). The a* value (red–green axis) increases with p-TMLs, indicating a shift towards a redder hue in samples like WB-TMA15 (5.82 ± 0.18) and WB-TMBM15 (6.73 ± 0.32). The b* value (yellow–blue axis) also shows variation, with WB-TMBM15 displaying the highest yellow component (30.4 ± 0.01) in the crumb. Enriching bread with p-TMLs leads to darker, redder, and more yellow crust and crumb, with the maximum changes typically seen with the highest p-TML concentration (15%). There is not a straightforward correlation between the darkness of the crust (lower L* values) and higher acrylamide concentrations. For example, WB-TMA10 and WB-TMBM5, which are among the lighter samples (higher L* values), have some of the lowest acrylamide levels, while WB-TMBM15, which is not the darkest, has the highest acrylamide concentration. In their study, Dessev et al. highlighted that the coloration of bread crust significantly impacts acrylamide formation during baking (without edible insects). Specifically, as the crust’s color darkens (with the total color difference reaching up to 20–25), the acrylamide concentration in the bread increases linearly. However, beyond this level of crust coloration, the concentration of acrylamide tends to plateau or even decrease slightly [68]. However, the aforementioned Burešová et al. study shows that adding insects to cultured bread reduces the acrylamide content compared to a control group [66].

The inclusion of p-TMLs in the bread-making process does not result in acrylamide concentrations that exceed the strict limits set by the EU, indicating that this practice is safe from the standpoint of acrylamide regulation. This could be reassuring for both consumers and producers considering the nutritional benefits of incorporating p-TMLs into bread recipes without compromising food safety standards concerning acrylamide.

Different concentrations and types of p-TMLs influence the sugar content in bread samples (Table 5). Sucrose remains undetectable in most samples but increases in WB-TMBM10 and WB-TMBM15, which contain higher concentrations of p-TMLs grown on brewer’s spent grain, suggesting that a higher p-TML content might influence sucrose stability or formation. This could be due to the fact that specific rearing conditions may impact the sucrose content or its metabolic processes within the bread. There is a clear increasing trend in the glucose content as the concentration of p-TMLs increases, especially in the TMBM series, which suggests that the types of p-TMLs and their concentration may enhance glucose formation or reduce its utilization in the bread-making process. In our previous study, Jankauskienė et al. determined that the glucose content in larvae was statistically significantly higher compared with the substrate (*p* < 0.001). Specifically, when reared on agar–agar gels and brewer’s spent grain, the glucose concentration accumulated to 3 ± 0.09 and 1.95 ± 0.11 g/100 g, respectively [31]. The highest glucose level was observed in WB-TMBM15 (0.983 ± 0.009 g/100 g).

The highest amount of fructose was found in the control bread (*p* ≤ 0.05). This variation could be influenced by differences in the metabolic pathways affected by p-TML type and concentration.

Maltose is composed of two glucose units, and it is less sweet and found in grains [69]. It is used less frequently in cooking but is important in brewing beer [70,71,72]. In our previous study by Jankauskienė et al., the maltose content in larvae was below the detection limit (<0.20 g/100 g) [31], which could have influenced the maltose levels in bread upon the addition of mealworm larvae grown under different conditions; however, no clear, statistically significant trends in the maltose content were observed in the larvae. The variations in the maltose concentration with different percentages of p-TMLs in bread samples can be explained by the interaction between the protein content and enzyme activity during bread making. Adding 5% p-TMLs likely increases the maltose content due to the presence of additional enzymes that break down starch into maltose. When the p-TML content increases to 10%, the higher protein levels may inhibit the enzyme activity, leading to a reduction in maltose levels. However, at 15% p-TMLs, the enzyme activity could either become more effective again, or the proteins may stabilize, resulting in an increase in the maltose concentration [73,74]. Conversely, in bread, the maltose content initially decreases with lower concentrations of p-TMLs but increases significantly in the sample with the highest concentration of p-TMLs (WB-TMBM15). This could explain why the highest amount of maltose detected is found in bread, whereas the highest concentration of grains is used in beer [70]. This trend may reflect the changes in enzymatic activities influenced by the larval content and type [75].

Proteins are vital in human nutrition, as they provide the amino acids necessary for tissue growth, maintenance, and regeneration [76,77]. Furthermore, using insects like *T. molitor* as a protein source is significantly more sustainable compared to traditional animal-derived protein sources [78]. Insect proteins can help diversify food sources and reduce dependence on traditional protein sources, which may be susceptible to supply disruptions due to climate change, diseases, or economic difficulties [79]. Additionally, innovation in food production can attract consumers looking for healthier or more interesting dietary alternatives [80].

The highest protein content in larvae cultivated under different conditions was found precisely on the same brewer’s grains used (59.18%), just as in our current study; this had a statistically significant effect on the protein content of the bread [31]. Since mealworms are a protein ingredient, the protein content increases progressively with the addition of p-TMLs in all enriched samples compared to the control (WB-C) [81,82]. The highest protein content is observed in WB-TMBM15 (12.3 ± 0.09%), suggesting that both the medium and higher percentage of larvae contribute significantly to protein enrichment. In a study conducted by Khuenpet et al., bread products with added larval-stage mealworm powder at 0, 5, 10, and 15% of the wheat flour content had statistically significantly increased protein contents of 9.63, 12.63, 13.21, and 13.73%, respectively [83].

It could be assumed that fat content, similar to protein, increases with a higher p-TML concentration. In our study [31], one of the highest amounts of fat was found in larvae grown on agar–agar gels (32.54 ± 0.02%). Analogous to this study, the most notable increase is in WB-TMA15 (4.09 ± 0.03%), indicating that agar–agar gels with a concentration of 15% larvae significantly enhance the fat content. These findings align with those of González et al. [84] and Osimani et al. [30], and it is recommended that the fat content of insect powder be modified (i.e., defatted) to produce a better-balanced enriched bread [29].

Acrylamide typically forms during the Maillard reaction, which involves reducing the sugar and amino acid levels. The correlation was not detected between higher glucose (0.983 g/100 g) and maltose (2.36 g/100 g) levels and increased acrylamide formation (30.4 μg/kg) in samples like WB-TMBM15 because the relationship does not appear consistent across all samples.

The inclusion of p-TMLs in bread significantly impacts the nutritional profile, increasing the protein and fat contents while also affecting the sugar levels. The impact is more pronounced with higher p-TML percentages and varies with the type of growth substrate used. WB-TMBM15 stands out as having the highest increases in maltose, protein, and fat, making it significantly different in terms of nutritional enhancement compared to the control and other enriched samples.

The addition of p-TMLs to bread significantly influences the composition of essential amino acids, with variations evident across different concentrations and rearing conditions (Figure 1, Appendix A). As the concentration of p-TMLs in bread increases, there is a marked enhancement in the levels of essential amino acids such as lysine, methionine, threonine, and tryptophan. For instance, the lysine content rises from 0.14 g/100 g in the control (WB-C) to 0.35 g/100 g in the sample with the highest concentration of p-TMLs (WB-TMBM15). This trend is consistent across other amino acids, showing a significant increase as the p-TML content increases, which indicates that p-TMLs are a good source of these vital nutrients. Roncolini et al., in their study, analyzed the addition of mealworm powder to bread, and the trends were similar: adding p-TMLs increased the total amount of amino acids and essential amino acids. In their investigation, among the essential amino acids, tyrosine, methionine, isoleucine, and leucine exhibited the highest average increases in breads fortified with 10% p-TMLs, containing 68%, 60%, 53%, and 46%, respectively [85]. In their study, Kowalski et al. analyzed the incorporation of different larvae into wheat bread, ranging from 10 to 30%, specifically *A. diaperinus*, *A. domesticus*, and *T. molitor* powders. The results of this investigation coincided with our results and demonstrated that the amino acid profile was significantly enriched in essential amino acids both in the insect powder and in the bread products into which they were incorporated compared to wheat flour and wheat bread [86].

The amino acid profiles also vary with different rearing conditions of the larvae, as seen with the TMA and TMBM samples. For example, the samples WB-TMBM15 and WB-TMA15 show different levels of certain amino acids, suggesting that the substrate on which the larvae are grown can influence their nutritional content and, subsequently, their impact on bread when used as an additive. In their research, Cozmuta et al. incorporated *A. domesticus* and yellow mealworm powders into functional bread [87]. According to the study, when compared with standard bread, bread containing 10% *A. domesticus* and 10% yellow mealworm demonstrated a significant increase in the essential amino acids valine (9.72%) and tyrosine (1.86%). In contrast, in our study, it was found that in TMBM15, tyrosine increased by 104.55% and valine by 90.48%. This disparity could be attributed to our use of freeze-dried powders, a process which, as research indicates, preserves a higher amount of amino acids [88,89].

A study about in vitro amino acid and protein bio-accessibility from edible insects with *A. diaperinus* and *T. molitor* larvae in bread was conducted by Igual et al., and the results demonstrated that bread incorporating pea protein, *A. diaperinus*, and *T. molitor* at 5% and 10% concentrations had significantly higher levels of essential and non-essential amino acids (12.170–16.274 mg TAA/100 g) compared to the control bread (10.843 mg TAA/100 g). Notably, the content of amino acids increased with higher concentrations of the experimental ingredients in the bread. Additionally, after in vitro digestion, the experimental bread showed a greater accessibility of amino acids and proteins compared to the control [90].

There is a clear, statistically supported trend showing that the inclusion of p-TMLs improves the overall amino acid profile of the bread. This enhancement could be indicative of an overall improvement in the bread quality, particularly in terms of its nutritional value, which is crucial for protein-rich diets. Considering the amino acid enrichment, WB-TMBM15 appears to be the best product among the listed samples. It not only has higher levels of almost all essential amino acids but also shows the most significant increase in its protein content, making it potentially the most nutritious option.

In conclusion, the enrichment of bread with p-TMLs significantly enhances its essential amino acid profile, particularly when higher concentrations of p-TMLs are used or specific rearing conditions are applied.

The FA profile of larvae can vary depending on their rearing conditions and processing methods (Table 6). This implies that different types of p-TMLs can differently affect the FA composition of the final product. p-TMLs are rich in unsaturated FAs, including monounsaturated fatty acids (MUFAs) and polyunsaturated fatty acids (PUFAs). The MUFA levels are higher in p-TML-enriched samples compared to the control, while the PUFA content was decreased with a higher percentage of mealworms incorporated in bread. WB-TMBM10 has the highest MUFA content at 45.54%, while WB-TMA15 shows the highest content of PUFAs at 44.39% compared to other bread samples with mealworms, indicating that a higher p-TML content can enrich bread with beneficial unsaturated fats. The addition of *T. molitor* larvae to bread significantly affects the FA composition across different types of FAs: there is a clear increase in C18:1 (oleic acid) in bread with p-TMLs added, peaking in samples with p-TMLs grown on brewer’s spent grain (WB-TMBM10). This indicates that p-TMLs can enrich bread with oleic acid, which is beneficial for heart health [91,92]. Other MUFAs like C16:1 show a decrease with an increased p-TML content, suggesting a specific influence of the larvae’s diet and processing on certain FA profiles.

There is a noticeable variation in the levels of specific saturated fatty acids (SFAs) with the inclusion of p-TMLs. The total SFA content varies across samples, with the lowest percentage in WB-TMA10 (17.06%) and the highest in WB-TMBM15 (30.86%). This variability implies that the type and amount of p-TMLs can influence the SFA levels in the bread. For example, palmitic acid (C16:0) generally decreases in the TMA samples as the larval content increases but shows a significant increase in the highest TMBM sample (WB-TMBM15). Stearic acid (C18:0) and other specific SFAs like C14:0 (myristic acid) and C17:0 show increased variability across samples, often increasing with higher percentages of larvae. The same results were found by Roncolini et al. [85], although compared to other insect powders, such as *A. diaperinus*, *A. domesticus*, and *T. molitor*, the C18:0 FA is significantly lower according to Kowalski [86].

The concentration of omega-3 FAs significantly increases with the percentage of *T. molitor* larvae. The control wheat bread (WB-C) has the lowest omega-3 content at 1.01%, while the highest concentration is found in WB-TMA15 (6.90%), indicating a positive correlation between the larval content and omega-3 levels. Our previous study, conducted by Jankauskienė et al., demonstrated that larvae reared on agar–agar gel exhibited the highest omega-3 FA content (6.99%) compared to other rearing conditions. This finding may influence the final product, explaining why the highest amount of larvae grown on agar contains the most of these acids [32].

The omega-6 content decreases in bread samples with larvae compared to the control, with the lowest values observed in WB-TMBM15 (27.22%). This suggests that the enrichment with p-TMLs tends to lower the omega-6 FA percentage. The same samples increased with omega-9 FAs, because in bread samples containing p-TMLs, WB-TMBM10 showed the highest level at 44.52%. This indicates that bread enrichment with *T. molitor* larvae can substantially boost the omega-9 content. The content of omega-6 FAs, particularly C18:2 w6 (linoleic acid), decreases with a higher p-TML content, which may suggest a nutritional shift towards a potentially more favorable omega-6-to-omega-3 ratio. In the aforementioned Roncolini et al. study, the results obtained support this hypothesis, as the amount of linoleic acid also decreases after the inclusion of mealworms, and, as a result, the ratio of omega 6/3 acids improves [85].

In summary, enriching bread with p-TMLs significantly alters its FA composition, generally increasing the levels of omega-3, omega-9, MUFAs, and PUFAs, while impacting the levels of omega-6 and SFAs. These changes suggest a potentially improved nutritional profile, particularly with higher proportions of omega-3 and reduced omega-6 FAs, which are beneficial for cardiovascular health. The type of medium and the percentage of p-TMLs used influence these outcomes markedly.

In Western culture, the inclusion of edible insects in food products, including bread, is not yet widespread, and attitudes towards this vary significantly by region and individual beliefs [93]. In many Western countries, insects are not a common food source, so the idea of eating them may provoke negative emotions [94,95]. This resistance could be tied to social norms and habits that have been formed over a long period [96,97]. However, there is a segment of consumers in the West who are open to new ideas and enjoy experimenting with unconventional food products [98,99]. Restaurants and food manufacturers targeting this market segment can introduce insects as an innovative ingredient, gradually changing consumer perceptions [100]. It should be noted that Western culture is gradually becoming more open to food products containing insects, particularly due to growing awareness of sustainability and healthcare aspects [101]. However, much work still needs to be conducted in the areas of education and marketing for this practice to become widely accepted.

The research study explored the emotional responses and overall acceptability of wheat bread samples enriched with varying percentages of p-TMLs, grown on different substrates (Table 7). The overall acceptability (OA) scores decreased as the percentage of p-TMLs increased in both TMA and TMBM samples, indicating potentially lower judges’ acceptability for higher p-TML contents. The WB-C sample had a high overall acceptability score of 93.07 and was predominantly associated with a consumer neutral emotion (0.718). The lowest emotions reported were happy (0.042) and surprised (0.0288). The valence was relatively low at 0.067. The WB-TM5A sample showed lower overall acceptability (81) compared to the control. Emotionally, it had a significantly higher happiness score (0.214) and a moderate increase in valence (0.102) compared to the control, suggesting a more positive emotional impact despite the lower acceptability. Our results are close to those of Gantner et al. because their study investigated the incorporation of p-TMLs (5%, 10%, and 15% levels replaced part of the wheat flour) into wheat bread, focusing on the effects of sensory characteristics. The sensory evaluations indicated that the amount of mealworm powder significantly affected the bread’s color, odor, flavor, and overall sensory quality, suggesting an optimal enrichment level at 5% to maintain the judges’ acceptability [102]. The lowest OA was observed in WB-TMBM15 at 48.21, possibly due to the higher p-TML content. However, opposite results were obtained by García-Segovia et al.; the researchers found that when the larvae were added, the acceptability of the bread increased [102]. García-Segovia et al., in their study, investigated the incorporation of p-TMLs (5%, 10%, and 15%) into wheat bread to examine consumer acceptance. The addition of mealworm powder at varying levels was found to improve the nutritional value by increasing the protein and fat contents of the bread. However, these additions also significantly affected the overall sensory profile. The results showed that the taste of all insect-enhanced bread was evaluated with significantly higher (*p* < 0.05) scores than the control and pea-enhanced bread. The bread with 10% *T. molitor* was the most appreciated for its visual appearance, aroma attribute, taste, and overall liking. Overall, the study supports the use of mealworm powder as a viable ingredient for enhancing bread with sustainable protein, though consumer hesitancy remains a significant barrier [103].

Sad, angry, surprised, scared, and contempt: these emotions varied across samples, but no clear trend was evident correlating with the amount of p-TMLs.

The emotions vary significantly with the composition of the larval substrate (agar–agar gels versus brewer’s spent grain) and the percentage added. This study illustrates that while innovative ingredients like p-TMLs can invoke curiosity and positive emotions in lower concentrations (as seen in WB-TM5A), higher concentrations may lead to decreased acceptability and more negative emotions, highlighting the challenges in consumer acceptance of unconventional food ingredients.

## 4. Conclusions

This study has demonstrated that incorporating freeze-dried p-TMLs at different concentrations (0%, 5%, 10%, and 15%) into wheat bread impacts both the nutritional quality and sensory characteristics of the final product. Our findings demonstrate that p-TMLs, particularly those reared on brewery spent grains, enrich bread with essential amino acids, protein, fat with monounsaturated FAs, and have a high polyunsaturated FA content, which are crucial for enhancing nutritional value. The acrylamide content within all formulations stayed well below the harmful thresholds, endorsing the safety of using p-TMLs in bread products under the studied conditions. While lower concentrations of p-TMLs (5%) are better received, higher percentages lead to a decrease in the sensory acceptability. This suggests that while mealworms can enhance the bread’s nutritional profile, their inclusion must be carefully calibrated to maintain consumer appeal. This research underscores the potential of p-TMLs as a sustainable and nutritious ingredient for bread making, although the balance between enhancing the nutritional content and maintaining consumer appeal remains a significant challenge. Future studies should continue to explore these dynamics, particularly focusing on consumer education and sensory optimization.

## Figures and Tables

**Table 1:** The proximal composition of *T. molitor* larvae grown under difference conditions.

Larvae	Moisture Content, g/100 g	Ash Content, g/100 g	Proteins, g/100 g	Fat, g/100 g	Carbohydrates, g/100 g	Total Content of Fiber, g/100 g	Fructose, g/100 g	Glucose, g/100 g
*T. molitor* larvae grown on wheat bran and agar–agar gel.	4.80 ± 0.54	3.08 ± 0.13	49.55 ± 0.05	32.54 ± 0.02	10.03 ± 0.51	5.5 ± 0.20	0.37 ± 0.04	3 ± 0.09
*T. molitor* larvae grown on brewer’s spent grain	7.43 ± 0.79	3.81 ± 0.16	59.18 ± 0.00	20.23 ± 0.02	9.34 ± 0.80	8.07 ± 0.35	0.56 ± 0.15	1.95 ± 0.11

**Table 2:** The ingredients added to the bread, as a percentage of flour weight.

	Ingredients					
	Water	Wheat Flour (550D)	Salt	Fresh Compressed Yeast	*T. molitor* Larvae Grown on Wheat Bran (TM_A_)	*T. molitor* Larvae Grown on Brewer’s Spent Grain (TM_BM_)
WB-C	56.0	100.0	1.5	3.0	-	-
WB-TM_5A_					5	-
WB-TM_A10_					10	-
WB-TM_A15_					15	-
WB-TM_BM5_					-	5
WB-TM_BM10_					-	10
WB-TM_BM15_					-	15

WB—wheat bread; C—control sample without additives; TM_A_—*Tenebrio molitor* larvae grown on agar–agar gels; TM_BM_—*Tenebrio molitor* larvae grown on brewer’s spent grain; 5, 10, and 15—the amount, in %, of added *Tenebrio molitor* larvae per 100 g of wheat flour, respectively.

**Table 3:** Influence of freeze-dried p-TMLs on wheat bread’s specific volume, porosity, shape coefficient, and mass loss after baking.

Bread Samples	Mass Loss after Baking, %		Porosity, %		Shape Coefficient			Specific Volume, cm^3^ g^−1^
WB-C	10.9 ± 1.2 ab		68.3 ± 1.2 d		1.47 ± 0.01 a			1.66 ± 0.10 a
WB-TM_5A_	10.9 ± 0.1 b		66.9 ± 0.8 d		1.46 ± 0.01 a			1.69 ± 0.13 a
WB-TM_A10_	9.59 ± 0.75 ab		64.5 ± 1.3 c		1.88 ± 0.02 b			1.60 ± 0.03 a
WB-TM_A15_	8.99 ± 0.26 a		59.1 ± 0.5 b		2.01 ± 0.04 c			1.53 ± 0.08 a
WB-TM_BM5_	8.68 ± 0.60 a		66.1 ± 1.0 cd		2.23 ± 0.04 d			1.63 ± 0.07 a
WB-TM_BM10_	9.65 ± 0.45 ab		60.2 ± 0.6 b		2.32 ± 0.01 e			1.72 ± 0.05 a
WB-TM_BM15_	11.2 ± 0.6 b		53.7 ± 1.3 a		1.99 ± 0.03 c			1.79 ± 0.05 a
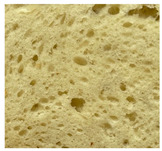	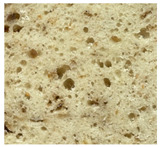			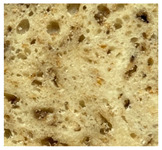			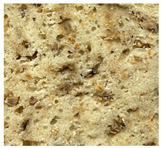	
WB-C	WB-TM_5A_			WB-TM_10A_			WB-TM_15A_	
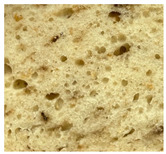		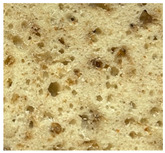				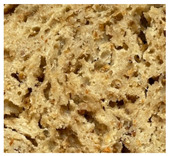		
WB-TM_5BM_		WB-TM_10 BM_				WB-TM_15 BM_		

Note: Data are expressed as mean values (*n* = 3) ± standard error (SE). a–d mean values within a row with different letters are significantly different (*p* ≤ 0.05). WB—wheat bread; C—control sample without additives; TM_A_—*Tenebrio molitor* larvae grown on agar–agar gels; TM_BM_—*Tenebrio molitor* larvae grown on brewer’s spent grain; 5, 10, and 15—the amount, in %, of added *Tenebrio molitor* larvae per 100 g of wheat flour, respectively.

**Table 4:** Acrylamide concentration and color coordinates of wheat bread enriched with freeze-dried p-TMLs.

Bread Samples	Crust			Crumb			Acrylamide Concentration, µg kg^−1^
	L*	a*	b*	L*	a*	b*	
WB-C	39.1 ± 0.6 a	12.9 ± 0.2 ab	15.6 ± 0.4 a	74.6 ± 0.6	2.38 ± 0.22 a	15.3 ± 0.3 a	27.0 ± 0.05 a
WB-TM_5A_	37.3 ± 0.5 a	12.4 ± 0.4 a	17.1 ± 0.2 a	70.8 ± 1.2	3.82 ± 0.18 b	16.1 ± 0.2 a	25.1 ± 0.04 b
WB-TM_A10_	49.7 ± 0.8 c	12.0 ± 0.2 a	21.4 ± 0.5 b	59.3 ± 0.6	3.93 ± 0.05 b	16.0 ± 0.4 a	21.2 ± 0.02 c
WB-TM_A15_	46.6 ± 0.3 b	12.8 ± 0.2 a	22.9 ± 0.2b c	56.3 ± 1.2	5.82 ± 0.18 c	15.6 ± 0.3 a	22.1 ± 0.04 d
WB-TM_BM5_	49.6 ± 0.4 c	14.0 ± 0.1 bc	24.8 ± 0.2 d	61.2 ± 0.2	3.36 ± 0.33 ab	16.6 ± 0.3 ab	21.2 ± 0.03 c
WB-TM_BM10_	43.5 ± 0.6 b	13.8 ± 0.2 bc	23.4 ± 0.3 cd	61.6 ± 0.4	4.09 ± 0.16 b	16.2 ± 0.3 a	23.5 ± 0.02 e
WB-TM_BM15_	44.4 ± 0.4 b	14.8 ± 0.3 c	22.8 ± 0.5 bc	58.4 ± 0.5	6.73 ± 0.32 c	17.9 ± 0.1 a	30.4 ± 0.01 f

Note: Data are expressed as mean values (*n* = 3) ± standard error (SE). a–f mean values within a row with different letters are significantly different (*p* ≤ 0.05). WB—wheat bread; C—control sample without additives; TM_A_—*Tenebrio molitor* larvae grown on agar–agar gels; TM_BM_—*Tenebrio molitor* larvae grown on brewer’s spent grain; 5, 10, and 15—the amount, in %, of added *Tenebrio molitor* larvae per 100 g of wheat flour, respectively.

**Table 5:** Sugar concentrations in bread samples enriched with freeze-dried p-TMLs, g/100 g.

Sugars	WB-C	WB-TM_5A_	WB-TM_A10_	WB-TM_A15_	WB-TM_BM5_	WB-TM_BM10_	WB-TM_BM15_
Lactose	<0.02	<0.02	<0.02	<0.02	<0.02	<0.02	<0.02
Galactose	<0.02	<0.02	<0.02	<0.02	<0.02	<0.02	<0.02
Sucrose	<0.02	<0.02	<0.02	<0.02	<0.02	0.050 ± 0.015	0.063 ± 0.003
Glucose	0.247 ± 0.003 a	0.443 ± 0.003 b	0.503 ± 0.001 c	0.647 ± 0.007 d	0.557 ± 0.003 e	0.603 ± 0.003 f	0.983 ± 0.009 g
Fructose	0.440 ± 0.006 a	0.407 ± 0.003 a	0.327 ± 0.002 b	0.317 ± 0.003 b	0.367 ± 0.012 c	0.343 ± 0.009 bc	0.317 ± 0.002 b
Maltose	1.43 ± 0.09 a	1.03 ± 0.01 bc	0.947 ± 0.003 b	1.18 ± 0.02 c	1.11 ± 0.01 bc	0.740 ± 0.021 d	2.36 ± 0.03 e
	%						
Protein content	8.32 ± 0.03 a	9.43 ± 0.08 b	10.6 ± 0.03 c	11.7 ±0.07 d	9.47 ± 0.12 b	11.0 ± 0.03 e	12.3 ± 0.09 f
Fat content	0.917 ± 0.002 a	2.06 ± 0.01 b	2.96 ± 0.02 c	4.09 ± 0.03 d	1.88 ± 0.01 e	2.90 ± 0.02 c	3.13 ± 0.01 f

Note: Data are expressed as mean values (*n* = 3) ± standard error (SE). a–g mean values within a row with different letters are significantly different (*p* ≤ 0.05). WB—wheat bread; C—control sample without additives; TM_A_—*Tenebrio molitor* larvae grown on agar–agar gels; TM_BM_—*Tenebrio molitor* larvae grown on brewer’s spent grain; 5, 10, and 15—the amount, in %, of added *Tenebrio molitor* larvae per 100 g of wheat flour, respectively.

**Table 6:** The FA compositions (as a percentage of the total FA content) in bread samples enriched with freeze-dried p-TMLs, %.

	WB-C	WB-TM_5A_	WB-TM_A10_	WB-TM_A15_	WB-TM_BM5_	WB-TM_BM10_	WB-TM_BM15_
SFAs							
C12:0	Nd	Nd	Nd	Nd	Nd	0.08 ± 0.001 a	0.02 ± 0.001 b
C14:0	Nd	0.81 ± 0.01 a	1.03 ± 0.02 b	1.08 ± 0.01 b	1.77 ± 0.02 c	1.46 ± 0.016 d	1.76 ± 0.002 c
C15:0	Nd	Nd	Nd	Nd	Nd	0.08 ± 0.001 a	0.18 ± 0.003 b
C16:0	19.13 ± 0.01 a	16.42 ± 0.08 b	13.32 ± 0.09 c	13.72 ± 0.04 d	20.15 ± 0.06 e	19.53 ± 0.04 f	24.56 ± 0.07 g
C17:0	Nd	Nd	Nd	Nd	0.07 ± 0.003 a	0.09 ± 0.002 b	0.23 ± 0.009 c
C18:0	4.06 ± 0.03 a	4.28 ± 0.05 b	2.65 ± 0.06 c	2.40 ± 0.03 d	3.49 ± 0.02 e	2.41 ± 0.01 d	4.10 ± 0.03 ab
C21:0	Nd	0.18 ± 0.001	Nd	Nd	Nd	Nd	Nd
C22:0	Nd	Nd	Nd	0.04 ± 0.002	Nd	Nd	Nd
C24:0	Nd	Nd	0.06 ± 0.001 a	0.20 ± 0.003 b	Nd	Nd	0.01 ± 0.001 c
MUFAs							
C16:1	1.74 ± 0.01 a	1.39 ± 0.02 c	1.24 ± 0.02 de	1.22 ± 0.01 d	1.37 ± 0.06 ce	0.96 ± 0.009 b	1.22 ± 0.03 d
C17:1	Nd	Nd	Nd	Nd	Nd	0.07 ± 0.001 a	0.01 ± 0.001 b
C18:1	25.17 ± 0.09 a	36.82 ± 0.08 b	37.38 ± 0.11 c	36.94 ± 0.07 cb	41.78 ± 0.12 d	44.52 ± 0.10 e	38.45 ± 0.08 f
C20:1	Nd	0.02 ± 0.001 a	Nd	nd	Nd	Nd	0.05 ± 0.003 b
C22:1	Nd	0.96 ± 0.007	Nd	nd	Nd	Nd	Nd
PUFAs							
C18:2 w6	48.90 ± 0.11 a	35.40 ± 0.09 b	38.26 ± 0.10 c	36.41 ± 0.09 d	29.35 ± 0.06 e	29.39 ± 0.07 e	26.76 ± 0.02 f
C18:3 α w3	1.01 ± 0.01 a	1.40 ± 0.01 b	1.28 ± 0.02 c	1.14 ± 0.01 d	1.13 ± 0.02 d	0.75 ± 0.002 e	0.93 ± 0.004 f
C20:2 w6	Nd	Nd	Nd	0.53 ± 0.003 a	Nd	0.07 ± 0.001 b	0.24 ± 0.005 c
C20:3 w3	Nd	2.32 ± 0.01 a	4.43 ± 0.03 b	5.77 ± 0.03 c	0.90 ± 0.004 d	0.58 ± 0.002 e	1.26 ± 0.01 f
C22:2 w6	Nd	Nd	0.35 ± 0.001 a	0.55 ± 0.002 b	Nd	Nd	0.22 ± 0.002 c
Omega-3	1.01 ± 0.051 a	3.72 ± 0.186 b	5.71 ± 0.286 c	6.90 ± 0.345 d	2.02 ± 0.101 ae	1.33 ± 0.067 ae	2.18 ± 0.109 e
Omega-6	48.9 ± 2.445 a	35.4 ± 1.770 bc	38.61 ± 1.931 b	37.48 ± 1.874 b	29.35 ± 1.468 bc	29.49 ± 1.475 bc	27.22 ± 1.361 c
Omega-9	25.17 ± 1.259 a	37.8 ± 1.890 b	37.38 ± 1.869 b	36.94 ± 1.847 b	41.78 ± 2.089 b	44.52 ± 2.226 b	38.5 ± 1.925 b
Total SFAs	23.19 ± 1.160 bc	21.69 ± 1.085 abc	17.06 ± 0.853 a	17.44 ± 0.873 ab	25.56 ± 1.274 cd	23.57 ± 1.183 c	30.68 ± 1.543 d
Total MUFAs	26.9 ± 1.345 a	39.19 ± 1.960 b	38.62 ± 1.931 b	38.16 ± 1.908 b	43.15 ± 2.158 b	45.54 ± 2.277 b	39.74 ± 1.987 b
Total PUFAs	49.91 ± 0.121 a	39.11 ± 0.110 bc	44.32 ± 0.150 ab	44.39 ± 0.135 ab	31.37 ± 0.084 c	30.81 ± 0.751 c	29.40 ± 1.220 c

Note: Nd—not detected. Data are expressed as mean values (*n* = 3) ± standard error (SE). a–g mean values within a row with different letters are significantly different (*p* ≤ 0.05). WB—wheat bread; C—control sample without additives; TM_A_—*Tenebrio molitor* larvae grown on agar–agar gels; TM_BM_—*Tenebrio molitor* larvae grown on brewer’s spent grain; 5, 10, and 15—the amount, in %, of added *Tenebrio molitor* larvae per 100 g of wheat flour, respectively.

**Table 7:** Overall acceptability and emotions induced in consumers with bread samples enriched with freeze-dried p-TMLs.

	OA	Neutral	Happy	Sad	Angry	Surprised	Scared	Disgusted	Con-tempt	Valence
WB-C	93.07 ± 2.29 a	0.718 ± 0.012 ab	0.042 ± 0.004 a	0.128 ± 0.063	0.078 ± 0.032	0.0288 ± 0.008 b	0.093 ± 0.015	0.104 ± 0.009	0.169 ± 0.108	0.067 ± 0.099
WB-TM_5A_	81.00 ± 2.93 abc	0.585 ± 0.016 ab	0.214 ± 0.005 c	0.072 ± 0.017	0.063 ± 0.003	0.029 ± 0.009 b	0.108 ± 0.054	0.096 ± 0.044	0.117 ± 0.036	0.102 ± 0.071
WB-TM_A10_	65.43 ± 1.86 cd	0.545 ± 0.042 ab	0.113 ± 0.003 ab	0.063 ± 0.013	0.086 ± 0.017	0.065 ± 0.011 b	0.093 ± 0.008	0.137 ± 0.012	0.121 ± 0.043	0.087 ± 0.064
WB-TM_A15_	62.79 ± 2.57 de	0.815 ± 0.043 a	0.045 ± 0.011 a	0.082 ± 0.016	0.014 ± 0.001	0.021 ± 0.006 b	0.054 ± 0.009	0.179 ±0.054	0.103 ± 0.032	0.004 ± 0.0001
WB-TM_BM5_	85.57 ± 4.00 ab	0.519 ± 0.072 b	0.138 ± 0.032 b	0.093 ± 0.024	0.063 ± 0.017	0.143 ± 0.066 ab	0.053 ± 0.019	0.083 ± 0.014	0.088 ± 0.009	0.119 ± 0.018
WB-TM_BM10_	75.43 ± 3.50 bcd	0.794 ± 0.087 ab	0.085 ± 0.007 ab	0.035 ± 0.009	0.013 ± 0.001	0.277 ± 0.054 a	0.138 ± 0.025	0.126 ± 0.028	0.107 ± 0.024	0.096 ± 0.029
WB-TM_BM15_	48.21 ± 4.14 e	0.590 ± 0.045 a	0.043 ± 0.008 a	0.173 ± 0.054	0.053 ± 0.032	0.038 ± 0.012 b	0.072 ± 0.013	0.029 ± 0.007	0.123 ± 0.022	−0.015 ± 0.012

Note: Data are expressed as mean values (*n* = 3) ± standard error (SE). a–e mean values within a row with different letters are significantly different (p ≤ 0.05). WB—wheat bread; C—control sample without additives; TM_A_—*Tenebrio molitor* larvae grown on agar–agar gels; TM_BM_—*Tenebrio molitor* larvae grown on brewer’s spent grain; 5, 10, and 15—the amount, in %, of added *Tenebrio molitor* larvae per 100 g of wheat flour, respectively.

## Data Availability

The raw data supporting the conclusions of this article will be made available by the author Agnė Jankauskienė on request.

## Appendix A

**Table A1:** Amino acid compositions in bread samples enriched with freeze-dried *T. molitor* larvae.

	WB-C	WB-TM_5A_	WB-TM_A10_	WB-TM_A15_	WB-TM_BM5_	WB-TM_BM10_	WB-TM_BM15_
g/100 g of dry matter							
Aspartic acid	0.34 ± 0.002 a	0.49 ± 0.001 b	0.56 ± 0.003 c	0.64 ± 0.008 d	0.49 ± 0.002 b	0.59 ± 0.001 e	0.68 ± 0.002 f
Glutamic acid	2.67 ± 0.01 ab	2.77 ± 0.02 ab	2.63 ± 0.01 a	2.61 ± 0.03 a	2.73 ± 0.04 ab	2.77 ± 0.05 ab	2.80 ± 0.04 b
Asparagine	0.00	0.00	0.00	0.00	0.00	0.00	0.00
Serine	0.37 ± 0.002 a	0.42 ± 0.001 b	0.44 ± 0.002 c	0.48 ± 0.003 d	0.44 ± 0.005 c	0.47 ± 0.001 d	0.53 ± 0.004 e
Glycine	0.25 ± 0.001 a	0.31 ± 0.001 b	0.35 ± 0.002 c	0.40 ± 0.003 d	0.33 ± 0.004 e	0.38 ± 0.002 f	0.45 ± 0.003 g
Histidine	0.17 ± 0.001 a	0.21 ± 0.001 bce	0.22 ± 0.002 cf	0.24 ± 0.003 d	0.20 ± 0.001 e	0.23 ± 0.002 df	0.27 ± 0.005 g
Threonine	0.33 ± 0.002 a	0.44 ± 0.003 b	0.47 ± 0.006 c	0.52 ± 0.007 d	0.43 ± 0.001 b	0.48 ± 0.002 c	0.55 ± 0.004 e
Alanine	0.23 ± 0.001 a	0.33 ± 0.004 b	0.39 ± 0.003 c	0.47 ± 0.004 d	0.35 ± 0.003 e	0.42 ± 0.002 f	0.53 ± 0.006 g
Arginine	0.24 ± 0.004 a	0.29 ± 0.001 bef	0.31 ± 0.002 cf	0.34 ± 0.004 d	0.28 ± 0.009 e	0.30 ± 0.008 cef	0.32 ± 0.001 cd
Proline	0.56 ± 0.001 a	0.53 ± 0.002 b	0.74 ± 0.009 c	0.62 ± 0.006 d	0.62 ± 0.001 d	0.64 ± 0.002 d	0.74 ± 0.004 c
Cystine	0.36 ± 0.001 a	0.35 ± 0.002 ab	0.34 ± 0.001 b	0.45 ± 0.002 c	0.45 ± 0.004 c	0.52 ± 0.002 d	0.65 ± 0.002 e
Tyrosine	0.22 ± 0.001 a	0.29 ± 0.001 b	0.33 ± 0.003 c	0.40 ± 0.002 d	0.30 ± 0.001 b	0.36 ± 0.002 e	0.45 ± 0.003 f
Valine	0.21 ± 0.001 a	0.27 ± 0.002 b	0.29 ± 0.003 c	0.33 ± 0.001 d	0.28 ± 0.002 bc	0.32 ± 0.002 d	0.40 ± 0.003 e
Methionine	0.10 ± 0.001 a	0.16 ± 0.001 b	0.18 ± 0.001 c	0.24 ± 0.003 d	0.23 ± 0.003 d	0.28 ± 0.005 e	0.30 ± 0.004 f
Lysine	0.14 ± 0.001 a	0.24 ± 0.002 b	0.26 ± 0.003 c	0.32 ± 0.001 d	0.22 ± 0.001 e	0.28 ± 0.001 f	0.35 ± 0.002 g
Isoleucine	0.23 ± 0.005 a	0.29 ± 0.002 b	0.31 ± 0.004 c	0.35 ± 0.002 d	0.29 ± 0.002 b	0.32 ± 0.003 c	0.37 ± 0.004 e
Leucine	0.50 ± 0.002 a	0.61 ± 0.003 b	0.63 ± 0.004 c	0.70 ± 0.003 d	0.60 ± 0.005 b	0.66 ± 0.002 e	0.76 ± 0.003 f
Phenylalanine	0.37 ± 0.001 a	0.41 ± 0.001 b	0.41 ± 0.002 b	0.43 ± 0.002 c	0.40 ± 0.005 b	0.43 ± 0.002 c	0.47 ± 0.002 e
Tryptophan	0.00	0.00	0.00	0.00	0.00	0.00	0.00
TOTAL	7.29 a	8.41 b	8.86 c	9.54 d	8.64 f	9.45 e	10.62 g

Note: Data are expressed as mean values (*n* = 3) ± standard error (SE). a–g mean values within a row with different letters are significantly different (*p* ≤ 0.05). WB—wheat bread; C—control sample without additives; TM_A_—*Tenebrio molitor* larvae grown on agar–agar gels; TM_BM_—*Tenebrio molitor* larvae grown on brewer’s spent grain; 5, 10, and 15—the amount, in %, of added *Tenebrio molitor* larvae per 100 g of wheat flour, respectively.
